# Whole-exome sequencing identifies FANC heterozygous germline mutation as an adverse factor for immunosuppressive therapy in Chinese aplastic anemia patients aged 40 or younger: a single-center retrospective study

**DOI:** 10.1007/s00277-023-05086-9

**Published:** 2023-01-09

**Authors:** Yingying Shen, Qi Liu, Hangchao Li, Wenbin Liu, Huijin Hu, Yuechao Zhao, Yuzhu Li, Ying Chen, Shan Liu, Qinghong Yu, Haifeng Zhuang, Liqiang Wu, Zhiping Hu, Zhiyin Zheng, Jianping Shen, Shenyun Lin, Yiping Shen, Yuhong Zhou, Baodong Ye, Dijiong Wu

**Affiliations:** 1 Department of Hematology, The First Affiliated Hospital of Zhejiang Chinese Medical University, #54 Youdian Road, Hangzhou, 310006 Zhejiang China; 2 National TCM Clinical Research Base (Hematology), Hangzhou, Zhejiang China; 3 The First School of Clinical Medicine, Zhejiang Chinese Medical University, Hangzhou, Zhejiang China; 4 Department of Clinical Evaluation Center, The First Affiliated Hospital of Zhejiang Chinese Medical University, Hangzhou, Zhejiang China

**Keywords:** Acquired aplastic anemia, *FANC*, Germline mutation, Immunosuppressive therapy, Hematopoietic stem cell transplantation

## Abstract

Acquired aplastic anemia (AA) is a bone marrow failure disorder characterized by pancytopenia, and immunosuppressive therapy (IST) is the optional first-line management. Several studies identified the influencing factors on IST response; however, there are still a considerable number of patients suffering from poor prognoses. In this study, we enrolled 61 AA patients aged ≤ 40 years old, and whole-exome sequencing (WES) found unexpected high *FANC* heterozygous germline mutations (28/61, 45.9%). Patients with *FANC* mutations have a significantly lower absolute reticulocyte count and CD34^+^ % in the bone marrow and also lower 3-, 6-, and 9-month IST response than that without mutation, which were 0% vs. 25% (*P* = 0.017), 26.3% vs. 42.1% (*P* = 0.495), and 29.4% vs. 72.2% (*P* = 0.011), especially in anti-thymocyte globulin combined with the cyclosporin A (ATG + CsA) group, which were 0% vs.33.4% (*P* = 0.143), 25% vs.83.3% (*P* = 0.103), and 25% vs. 100% (*P* = 0.003), respectively. The event-free survival in the *FANC*^wt^ group was also better than that in the *FANC*^mut^ group (*P* = 0.016) and also showed in patients who received ATG + CsA treatment (*P* = 0.045). In addition, all the adverse effects of *FANC* germline mutation were not significant in stem cell–transplanted group. Our result indicated that the WES-based detection of *FANC* heterozygous germline mutations may have a great meaning in predicting IST response of acquired AA. This study was registered at chictr.org.cn (# ChiCTR2100054992).

**Supplementary Information:**

The online version contains supplementary material available at 10.1007/s00277-023-05086-9.

## Introduction

Acquired aplastic anemia (AA) is a bone marrow disorder pathologically characterized by empty bone marrow and thus pancytopenia [1]. Immunosuppressive therapy (IST) and hematopoietic stem cell transplantation (HSCT) are the first-line treatments for AA. Despite a good response rate of 70–90% in AA [2], a great number of younger patients (≤ 40 years of age) is still hesitant for HSCT therapy due to the risk of treatment-related mortality and chronic and refractory complications, as well as unavailable donors. In China, over 70% of AA patients received IST as the first-line treatment, and the response rates in severe AA, non-severe AA, and transfusion-dependent AA patients were reported 60.6%, 64.3%, and 69.4%, respectively [3], denoting that approximately 40% of patients were unresponsive to IST. Several studies identified the influencing factors for varying IST response and survival, and the factors included patient age, disease severity, absolute reticulocyte (Ret) count (ARC), and the interval between diagnosis and treatment [4–6]. However, the overall response was never over 90%, which prompted us to further explore the underlying reasons.

With the advent of next-generation sequencing (NGS), new molecular insights into the pathogenesis of AA have been achieved. Some genetic mutations detected by NGS such as *DNMT3A*, *ASXL1*, *TP53*, *RUNX1*, and *CSMD1* were related to lower response to IST and poor overall and progression-free survival (PFS), while mutations on *PIGA* and *BCOR/BCORL1* were correlated with a better outcome. These elucidations in gene mutations may guide treatment of AA patients [7]. Whole-exome sequencing (WES), a new approach designed to sequence protein-coding regions of the genome that account for 1% of the genome [8], opened the door for identifying potential pathogenic genetic variants without spending the considerable resources required for the entire genome analysis. Up to date, a limited report is available on the use of WES in exploring the etiology and pathogenesis of AA. The WES is an accurate molecular technique to rule out congenital hematopoietic failures in young patients with pancytopenia. In our clinical center, we use the WES technique in younger patients suspected of AA if they can afford the expense. We found unexpected high *FANC* mutations in patients with AA (~ 50%), compared with 0.3–0.5% reported in the general healthy population [9]. Previous studies found that loss of function of any of the *FANC* genes may affect the expression of *FANC* protein and further influence the stability of hematopoietic stem cell chromosomes resulting in progressive bone marrow dysfunction and sensitivity to DNA cross-linking agents [10]. In addition, the intrinsic defects of hematopoietic stem cells in FA patients may cause B cells to be in long-term exposure to the harmful pro-apoptotic marrow environment, which was associated with decreased systemic immune function. But the effect of *FANC* heterozygous germline mutation on AA remained to be elaborated. In this study, we aimed to explore the significance of *FANC* heterozygous germline mutation in response to IST or HSCT and prognosis in AA patients.

## Methods

### Study design

The data of newly diagnosed AA patients in the First Affiliated Hospital of Zhejiang Chinese Medical University from January 2019 to July 2021 were retrospectively reviewed. The diagnosis and assessment of the disease were confirmed based on the Camitta criteria [11] and 2015 BJH guidelines [12], and the cases included severe aplastic anemia (SAA), very severe aplastic anemia (VSAA), non-severe aplastic anemia (NSAA), and transfusion-dependent none-severe aplastic anemia (TD-NSAA). Informed consent was obtained according to the Helsinki Declaration. This study was also registered at chictr.org.cn (# ChiCTR2100054992).

### Patient selection

Inclusion criteria were (1) patients younger than 40 years old; (2) patients diagnosed with AA; (3) patients who had a WES analysis result; and (4) patients who were negative for chromosomal breakage test.

Exclusion criteria were as follows: patients who had other or secondary pancytopenia, including paroxysmal nocturnal hemoglobinuria (PNH), myelodysplastic syndrome (MDS), hypocellular MDS, hypo-proliferative acute myeloid leukemia, and myelofibrosis, were excluded based on comprehensive differentiation diagnosis, including bone marrow biopsy/smear, bone marrow CD34^+^ ratio, and flow cytometry (FCM) analysis as well as NGS results.

### Baseline characteristics of patients

The baseline clinical characteristic of patients included age, gender, severity of disease, disease course defined as the interval time from diagnosis to treatment, treatment history, blood transfusion, routine blood test, and CD34^+^ % in bone marrow cells. Information on outcome variables was also retrieved for analysis.

### WES and data analysis

Bone marrow or peripheral blood was collected from AA patients, and WES was performed to check the *FANC* family (*FANCA*, *FANCB/FAAP95*, *FANCC*, *FANCD1/BRCA2*, *FANCD2*, *FANCE*, *FANCF*, *FANCG/XRCC9*, *FANCI*, *FANCJ/BRIP1*, *FANCL*, *FANCM*, *FANCN/PALB2*, *FANCO/RAD51C*, *FANCP/SLX4*, *FANCQ/ERCC4*, *FANCR/RAD51*, *FANCS/BRCA1*, *FANCT/UBE2T*, *FANCU/XRCC2*, *FANCV/REV7*, and *FANCW/RFWD3*) mutation status. All the mutation was further confirmed as germline mutation (heterozygous) with rechecking of the oral mucosa sample by means of PCR sequencing–based typing (PCR-SBT). The procedure for WES sequencing and data analysis was as follows.

### DNA extraction and detection

Genomic DNA extracted from the bone marrow or peripheral blood for each sample was fragmented to an average size of 180~280 bp and subjected to DNA library creation using established Illumina paired-end protocols. The Agilent SureSelect Human All ExonV6 Kit (Agilent Technologies, Santa Clara, CA, USA) was used for exome capture according to the manufacturer’s instructions. The Illumina NovaSeq 6000 platform (Illumina Inc., San Diego, CA, USA) was utilized for genomic DNA sequencing to generate 150-bp paired-end reads with a minimum coverage of 10× for ~ 99% of the genome (mean coverage of 100×).

### Data analysis

After sequencing, basecall file conversion and demultiplexing were performed with bcl2fastq software (Illumina). The resulting fastq data were submitted to in-house quality control software for removing low-quality reads and then were aligned to the reference human genome (hg19) using the Burrows-Wheeler Aligner (bwa) [13], and duplicate reads were marked using sambamba tools [14]. SNP/INDEL calling: single nucleotide variants (SNVs) and indels were called with SAMtools to generate gVCF [15]. The raw calls of SNVs and INDELs were further filtered with the following inclusion thresholds: (1) read depth > 4; (2) root-mean-square mapping quality of covering reads > 30; and (3) the variant quality score > 20.

### Annotation

Annotation was performed using ANNOVAR [16]. Annotations included minor allele frequencies from public control data sets as well as deleteriousness and conservation scores enabling further filtering and assessment of the likely pathogenicity of variants.

### Rare variant filtering

Filtering of rare variants was performed as follows: (1) variants with a MAF less than 0.05 in 1000 genomic data (1000g_all) [17], esp6500siv2_all [18], and gnomAD data (gnomAD_ALL and gnomAD_EAS) [19]. (2) Only SNVs occurring in exons or splice sites (splicing junction 10 bp) are further analyzed since we are interested in amino acid changes. (3) Then, synonymous SNVs which are not relevant to the amino acid alternation predicted by dbscSNV are discarded. The small fragment non-frameshift (< 10 bp) indel in the repeat region defined by RepeatMasker is discarded. (4) Variations are screened according to scores of SIFT [20], Polyphen [21], MutationTaster [22], and CADD [23] softwares. The potentially deleterious variations are reserved if the score of more than half of these four softwares supports harmfulness of variations [24]. Sites (> 2 bp) that did not affect alternative splicing were removed.

### ACMG classification

In order to better predict the harmfulness of variation, the classification system of the American College of Medical Genetics and Genomics (ACMG) was used. The variations are classified into pathogenic, likely pathogenic, uncertain significance, likely benign, and benign [25].

### Immunological index evaluation

On WES analysis, we found that besides *FANC* mutations, some patients also had other mutations that may affect the immune system. The serum immunoglobulins and lymphocyte subsets in peripheral blood including immunoglobulin A (IgA), IgG, IgM, CD4^+^CD25^+^CD127^−^Tregs, CD3^+^CD4^+^ T helper cells (Th), CD3^+^CD8^+^ cytotoxic T cells (Tc), the ratio of CD4^+^/CD8^+^, and CD19^+^B lymphocytes were collected and analyzed.

### Treatment strategies

The selection of treatment was based on the severity of the disease, age, and availability of donors as well as economic status, and all the decision was made with fully informed consent. For patients diagnosed with TD-NSAA, the standard management had no consensus yet. For patients who showed high value in obtaining a higher response, the HSCT and intensive IST were recommended. For patients who cannot afford the expense, cyclosporin (CsA) single treatment or supportive management was performed.

### CsA single treatment

The oral dose of CsA was 3–5 mg/kg/day, with a general target blood concentration (valley concentration) of 150–250 μg/L for adults, or 100–150 μg/L for children. The clinical dosage of CsA could be adjusted based on the drug concentration and curative effect.

### ATG combined with CsA

The dose of rabbit ATG (Thymoglobulin, Sanofi, France) was 3–4 mg/kg/day, and the ATG was used continuously for 5 days. Adrenal glucocorticoid was administered simultaneously when ATG was used and gradually withdrawn 14 days later to prevent allergic reaction as well as serologic reaction. The CsA was initiated on the first day when ATG was started, and the dose was adjusted according to the target drug concentration.

### HSCT

In our cohort, patients received allogeneic-HSCT (allo-HSCT) from HLA-identical sibling donor (ISD), matched unrelated donor (MUD), or haploidentical donor (HID). The conditioning regimen included cyclophosphamide of 30–50 mg/kg/day for 4 days, fludarabine of 30 mg/m^2^ for 5 days, and rabbit ATG (Thymoglobulin, Sanofi, France) of 2.5 mg/kg/day for 4 days. CsA, mycophenolate mofetil (MMF), and methotrexate (MTX) were administered as GVHD prophylaxis regimens.

### Efficacy assessment

Treatment efficacy was assessed at 3^rd^, 6^th^, and 9^th^ months after treatment initiation. All the clinical data were collected, and the baseline comparison was carried out. The patients who were lost to follow-up or treated for less than 3 months were not included in the efficacy evaluation. The treatment response in SAA was defined as follows [12]: (1) complete response (CR), hemoglobin concentration normal for age and gender, ANC count > 1.5 × 10^9^/L, and PLT count > 150 × 10^9^/L; (2) partial response (PR), transfusion independent, and no longer meeting criteria for severe disease; (3) no response (NR) and still severe. The treatment response in NSAA was defined as follows: (1) CR, the same criteria as for severe disease; (2) PR, transfusion-independent (if previously required) or doubling or normalization of at least one lineage (in line with any one of the CR criteria) or increase above baseline: hemoglobin of 30 g/L (if initially < 60), ANC of 0.5 × 10^9^/L (if initially < 0.5), PLT of 20 × 10^9^/L (if initially < 20); and (3) NR, worse or not meeting the criteria.

### Statistical analysis

The IBM SPSS 23.0 software was used for data analysis. The Shapiro-Wilk test was utilized to evaluate the normality of quantitative data. Quantitative data with normal distribution were assessed by a parametric test and those with non-normal distribution by a non-parametric test. Categorical data were assessed by Fisher’s exact test. Quantitative data conforming to normal distribution were represented as mean ± standard deviation (SD), and those with skewed distribution as median and 1^st^–3^rd^ quartiles. The enumeration data described the number of cases and percentages (%). Survival curves were constructed using the Kaplan-Meier method, and the log-rank test was used to determine differences between survival proportions. The *P* < 0.05 was considered statistically significant.

## Results

### Patients’ characteristics

#### Characteristic analysis of entire population

**Fig. 1:**
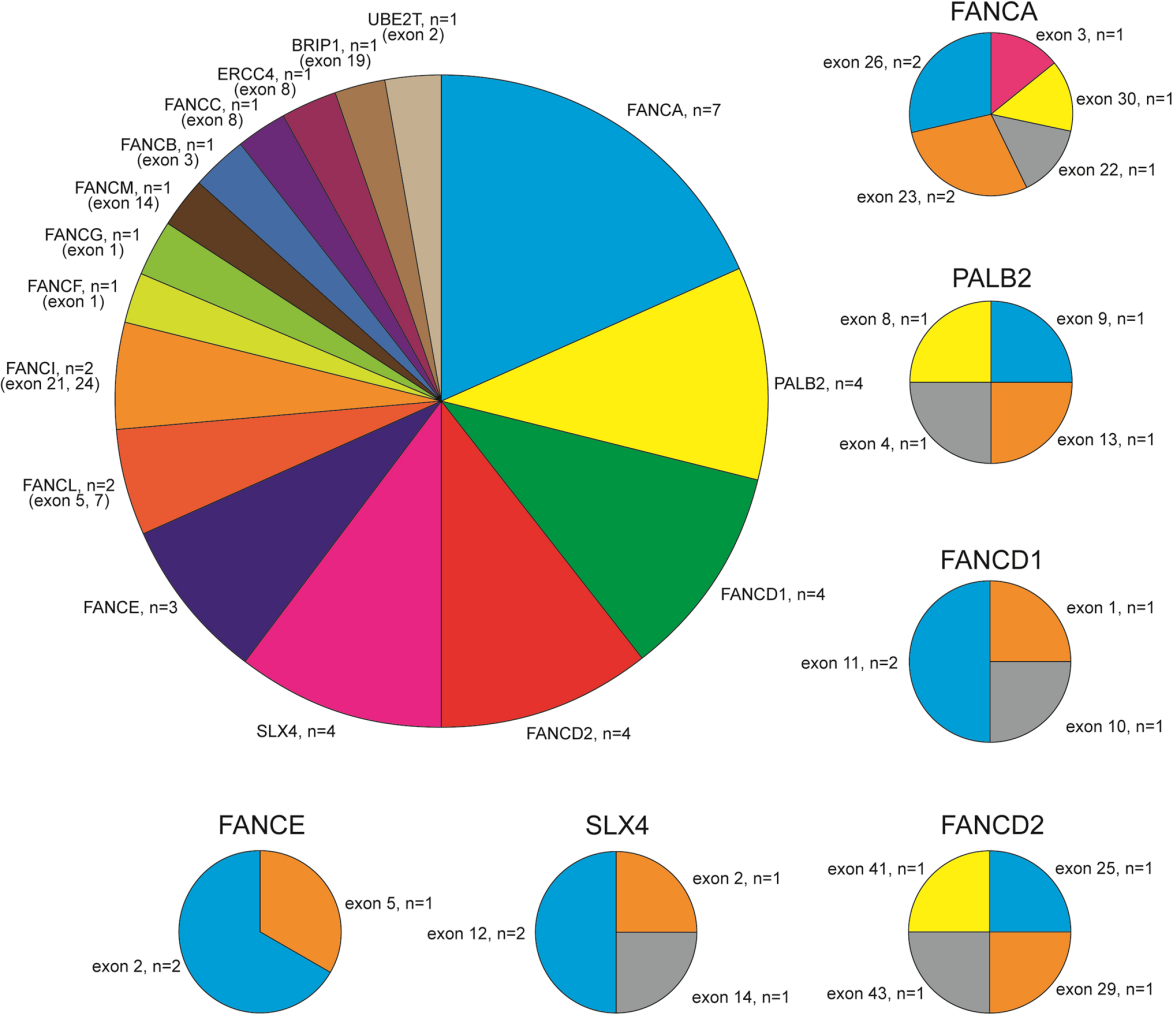
Frequencies and sites of FANC mutations. For the aplastic anemia patients with FANC mutations, all the specific mutated exon sites and frequencies were showed in the figure

A total of 61 patients diagnosed with AA and who underwent WES analysis were enrolled in this study. There were 27 females and 34 males with a median age of 23 (9–40) years. According to the severity of the disease, there were 9 cases of very severe AA (VSAA), 16 patients with severe AA (SAA), 28 patients with transfusion-dependent non-severe AA (TD-NSAA), and 8 cases of non-severe AA (NSAA). The rate of *FANC* germline mutations in our center accounts for 45.9% (28/61), and all the mutations were heterozygous. Detailed clinical data of patients with gene mutation are shown in Supplemental Table 1. The frequencies of *FANC* mutant genes were 7/38 for *FANCA*, 4/38 for *PALB2* (*FANCN*), 4/38 for *FANCD1*, 4/38 for *FANCD2*, 4/38 for *SLX4* (*FANCP*), 3/38 for *FANCE*, 2/38 for *FANCL* and *FANCI*, and 1/38 for *FANCF*, *FANCG*, *FANCM*, *FANCB*, *FANCC*, *ERCC4* (*FANCP*), *BRIP1* (*FANCJ*), and *UBE2T* (*FANCT*) (Fig. 1).

#### Characteristic analysis based on FANC status

To better understand the influence of *FANC* status on the baseline characteristics of the AA, the patients were divided into two groups based on *FANC* mutation status, AA with wide-type *FANC* (*FANC*^wt^) and AA with mutated *FANC* (*FANC*^mut^). The baseline clinical characteristics, such as age, gender, the severity of disease, disease course, treatment history, blood transfusion, peripheral hemogram, and absolute reticulocyte count, as well as CD34^+^ % of bone marrow cells, were compared, and all the indexes were similar across the subgroups (Table 1). However, the absolute reticulocyte count (ARC) and CD34^+^ % of bone marrow were found remarkably lower in the *FANC* mutation group (*P* = 0.021, 0.015, respectively). In addition, there were also more VSAA and SAA cases in the *FANC*^mut^ group (14/28) when compared to the *FANC*^wt^ group (11/33), but not statistically significant (*P* = 0.205).

**Table 1:** Comparison of the clinical features of AA patients with/without FANC mutation

	AA with FANC mutation (28, 45.9%)	AA without FANC mutation (33, 54.1%)	*X*^2^/*Z*	*P*
Age/median (range)	20.5 (9–38)	28 (14–40)	− 2.615	**0.009**
Gender/female (%)	13 (46.43%)	14 (42.42%)	0.098	0.754
Age with hematology abnormality/median (range)	17.5 (3–38)	19 (3–40)	− 1.232	0.218
Transfusion dependent/cases (%)	26 (92.86%)	27 (81.82%)	0.796	0.372
Classification (disease severity)/(cases, %)			3.275	0.351
VSAA	6 (21.42%)	3 (9.09%)		
SAA	8 (28.57%)	8 (24.24%)		
TD-NSAA	12 (42.86%)	16 (48.48%)		
NSAA	2 (7.14%)	6 (18.18%)		
Disease course (months)/median (range)	1 (0.25–2.75)	1 (0.25–4.75)	− 0.270	0.787
WBC count (10^9^/L)/median (range)	2.05 (0.2–4.2)	2.7 (0.5–5.8)	− 1.818	0.069
Absolute neutrophil count (10^9^/L)/median (range)	0.7 (0.01–1.7)	1.0 (0–3)	− 1.487	0.137
Neutrophil < 0.5 * 10^9^/L/cases (%)	10 (35.71%)	9 (27.27%)	0.503	0.478
RBC count (10^9^/L)/median (range)	1.770 (0.69–4.55)	1.88 (1.16–4.0)	− 1.129	0.259
HB (g/L)/median (range)	56.5 (28–102)	60 (38–124)	− 1.644	0.100
PLT count (10^9^/L)/median (range)	11 (1–36)	15 (3–68)	− 1.247	0.213
Absolute Ret count (10^9^/L)/median (range)	2.465 (0.02–7.38)	3.12 (0.3–11.23)	− 2.316	**0.021**
Total serum immune globulin (g/L)/median (range)	19.9 (13.8–32.2)	22 (12.1–34.5)	− 1.083	0.279
IgA	1.66 (0.72–2.64)	1.805 (0.51–17.3)	− 0.202	0.840
IgG	8.08 (6.4–14.9)	10 (5.67–15.1)	− 1.455	0.146
IgM	1.28 (0.39–2.49)	1.01 (0.18–3.9)	− 1.029	0.303
CD3^+^CD45^+^ (%)/median (range)	78.59 (58.70–88.85)	77.055 (62.22–91.26)	− 0.431	0.666
CD3^+^CD4^+^ (% of CD3^+^CD45^+^)/median (range)	38.30 (9.93–56.30)	39.07 (3.93–60.52)	− 0.634	0.526
CD3^+^CD8^+^ (% of CD3^+^CD45^+^)/median (range)	34.445 (17.41–63.15)	31.985 (16.67–81.07)	− 0.713	0.476
CD4^+^CD25^+^CD127^−^ (% of CD4^+^CD25^+^)/median (range)	5.55 (2.5–12.3)	5.05 (2.4-8.2)	− 0.845	0.398
CD4^+^/CD8^+^ (ratio)/median (range)	1.137 (0.157–3.234)	1.198 (0.048–3.032)	− 0.722	0.470
CD19^+^ (% of total lymphocytes/median (range)	13.37 (1.63–31.48)	11.04 (0–29.33)	− 0.476	0.634
BM CD34^+^ (% of alive cells)/median (range)	0.055 (0.01–0.08)	0.13 (0.03–0.60)	− 2.436	**0.015**

Values in bold indicate *P*<0.05

*VSAA* very severe aplastic anemia, *SAA* severe aplastic anemia, *TD-NSAA* transfusion-dependent non-severe aplastic anemia, *NSAA* non-severe aplastic anemia, *WBC* white blood cell, *RBC* red blood cell, *HB* hemoglobin, *PLT* platelet, *BM* bone marrow

### Treatment distribution

**Fig. 2:**
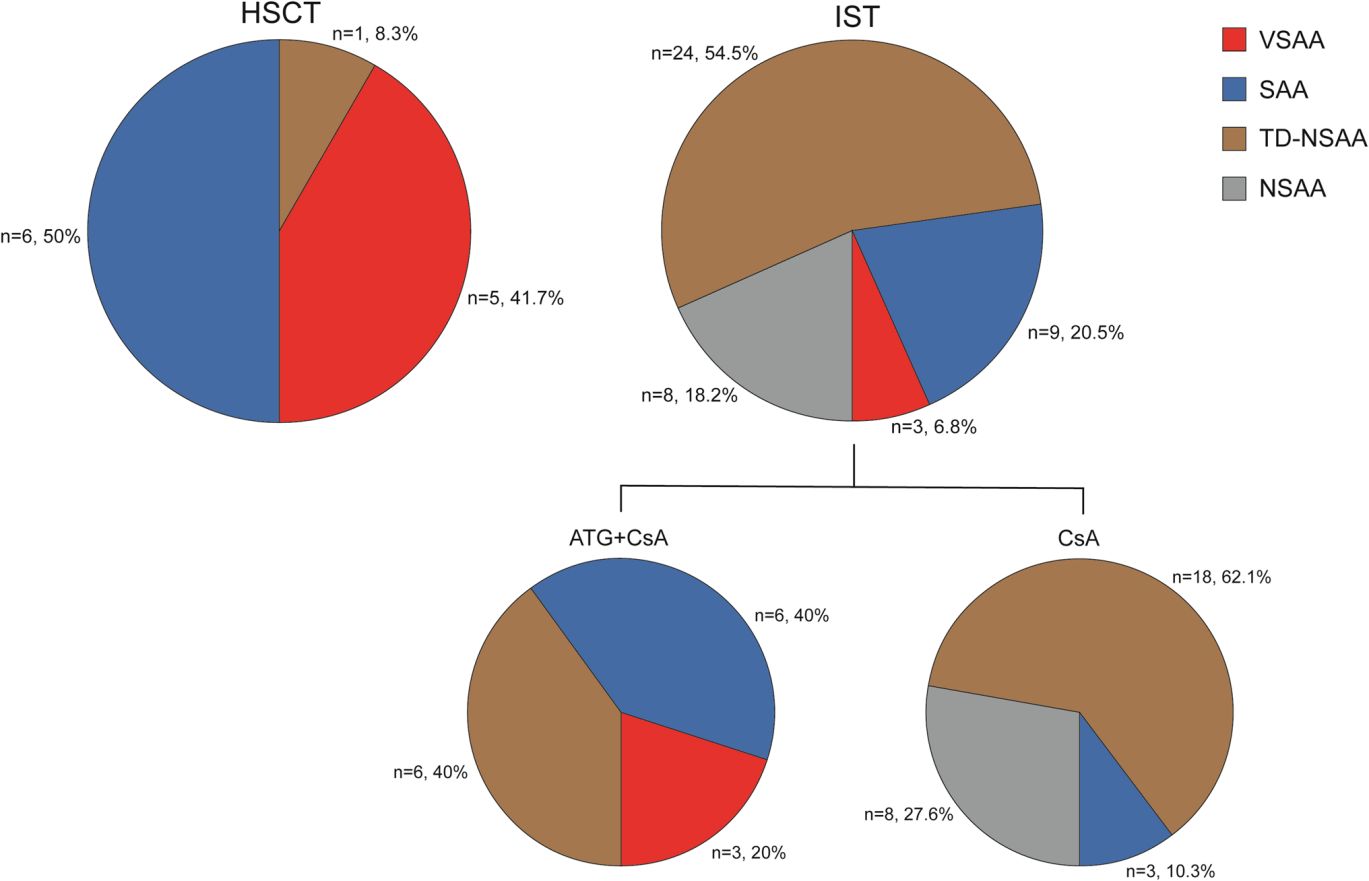
Distribution of disease severity in HSCT and IST (CsA±ATG)

**Fig. 3:**
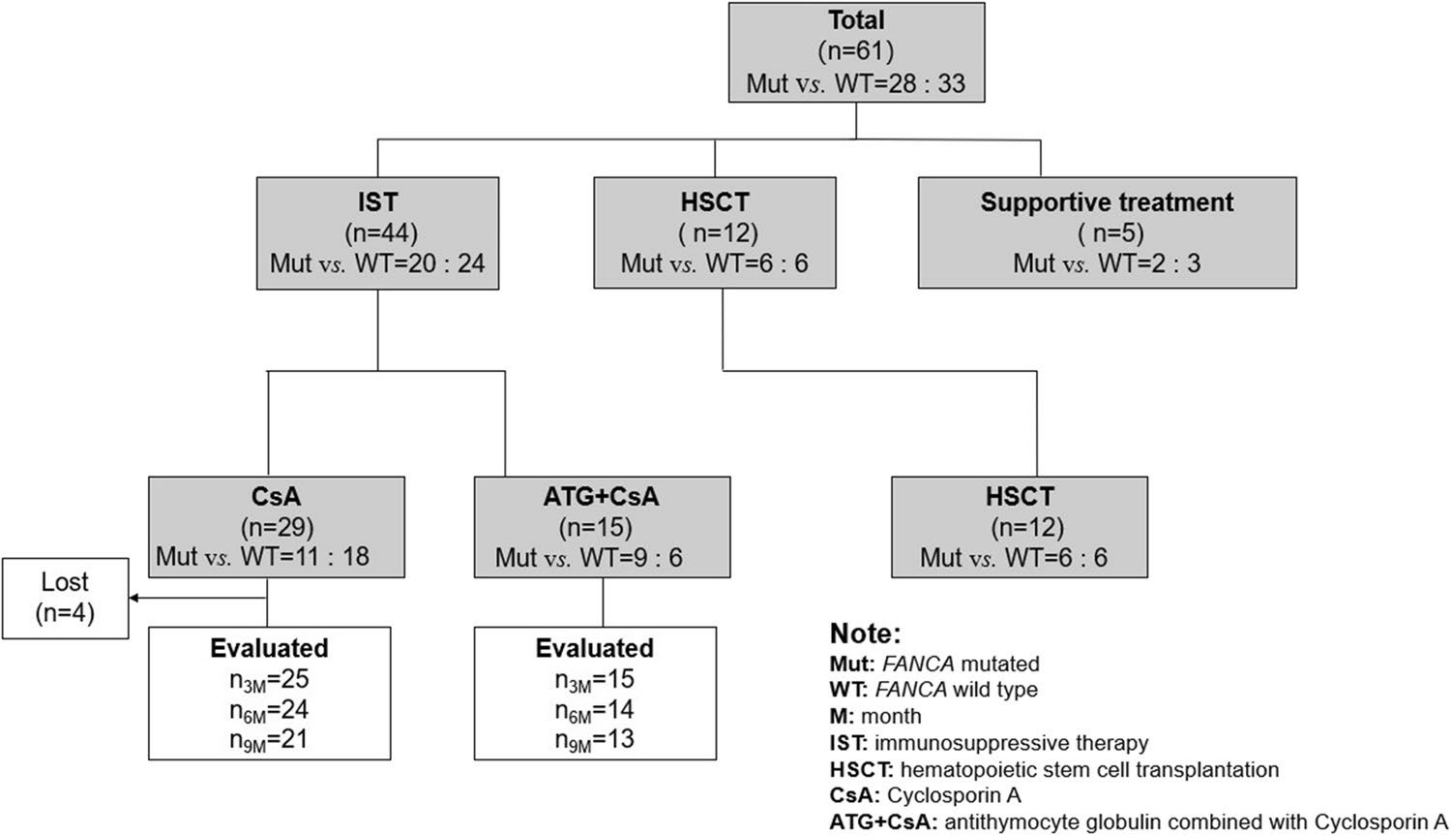
Treatment distribution of all the AA patients enrolled. All the treatment regimens were described

For all 61 patients enrolled in the analysis, the treatment regimens were selected based on the severity of the disease and patients’ preferences (Fig. 2). Forty-four patients initially received IST (29 patients with CsA single treatment and 15 patients with ATG + CsA), 12 patients firstly underwent HSCT (including 3 from 10/10 MUD, and 9 from HID), and the remaining 5 patients only received supportive treatment. During the observation, in the CsA single treatment group (*n* = 19), 4 patients were lost to follow-up within 3 months (Fig. 3).

### Response comparison in the entire population

**Fig. 4:**
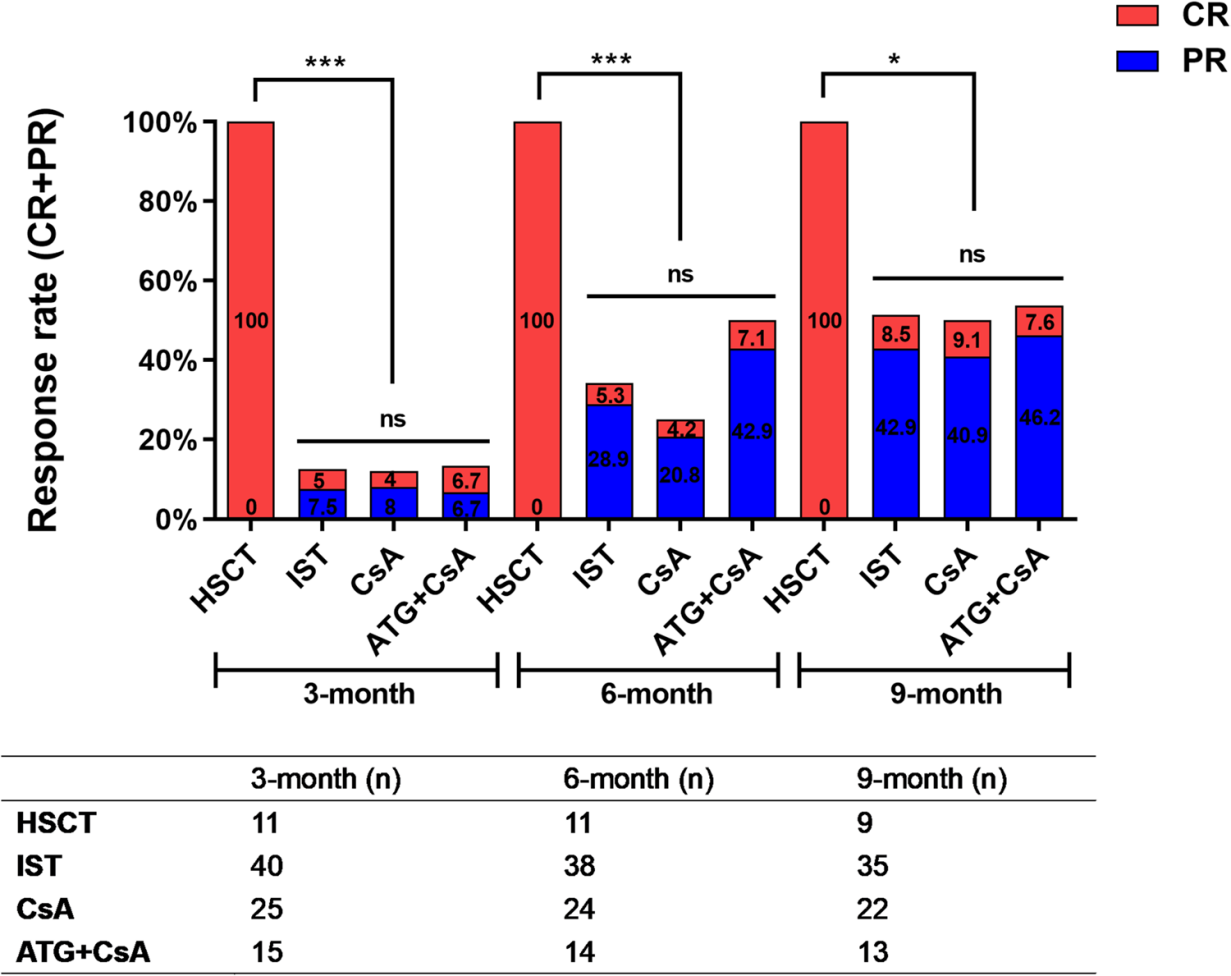
Response comparison in the entire population. The response rate (CR+PR) of each regimen (HSCT, IST, CsA or ATG+CsA) were compared 3, 6, and 9 months after treatment. ns means no significant difference, ***<0.001, *<0.05. The number of patients included in each subgroup were shown

There were 25, 15, and 12 patients who were eligible for efficacy analysis in the CsA single treatment, ATG + CsA, and HSCT groups, respectively. The overall hematological response in patients who received HSCT at 3^rd^, 6^th^, and 9^th^ months was all 100%, compared to 12.0%, 25.0%, and 50.0% in the CsA single treatment group and 13.4%, 50%, and 53.8% in the ATG + CsA group, respectively. The combined IST efficiency at the 3^rd^, 6^th^, and 9^th^ month was 12.5%, 34.2%, and 51.4%, respectively. The were no significant differences between the CsA single treatment and ATG + CsA groups at each time point, and the efficiency of HSCT was superior to IST, regardless of the CsA group or ATG + CsA group at any time points (3^rd^, 6^th^, and 9^th^ months, all *P* < 0.01) (Fig. 4).

### Subgroup efficacy analysis

#### Response rate of entire population based on FANC germline mutations

**Fig. 5:**
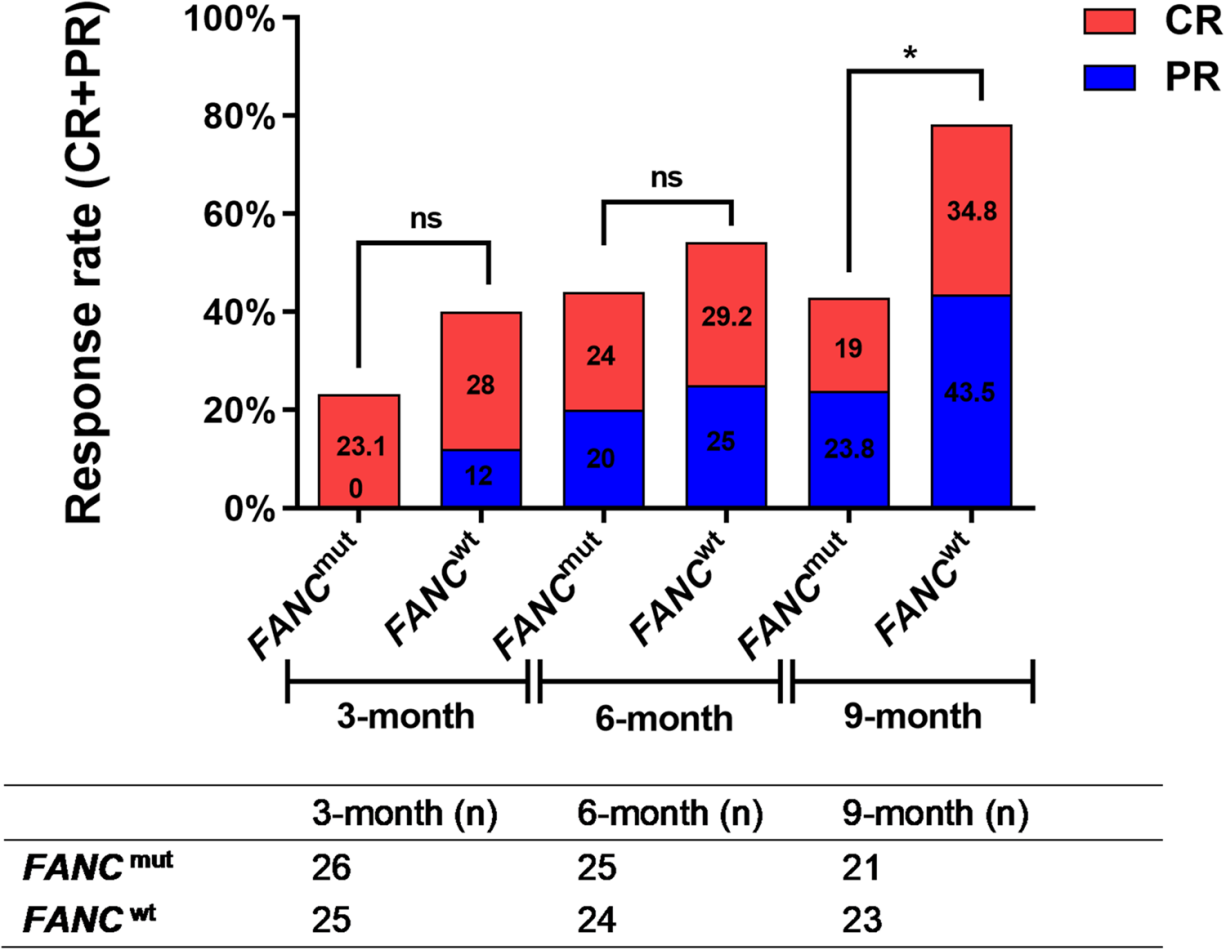
Response rate of entire population based on FANC germline mutations. The response rate (CR+PR) of aplastic anemia patients with or without FANC mutation were compared based on 3, 6, 9 months observation. ns means no significant difference, *<0.05. The number of patients included in each subgroup were shown

To assess the effect of *FANC* germline mutation on therapy response, patients were divided into subgroups based on *FANC* status, and the distribution of therapeutic methods was similar between groups (Supplemental Table 2). The overall hematological response rates in patients with *FANC* mutation at 3^rd^, 6^th^, and 9^th^ months were 23.1%, 44.0%, and 42.8%, respectively, compared to 40.0%, 54.2%, and 78.3% in the *FANC* non-mutation subgroup, and significant differences were found at the 9^th^ month (*P* = 0.029), while no differences were found at the 3^rd^ and 6^th^ (*P* = 0.237 and 0.572, respectively) (Fig. 5).

#### Comparison of IST vs. HSCT on response rate based on FANC status

**Fig. 6:**
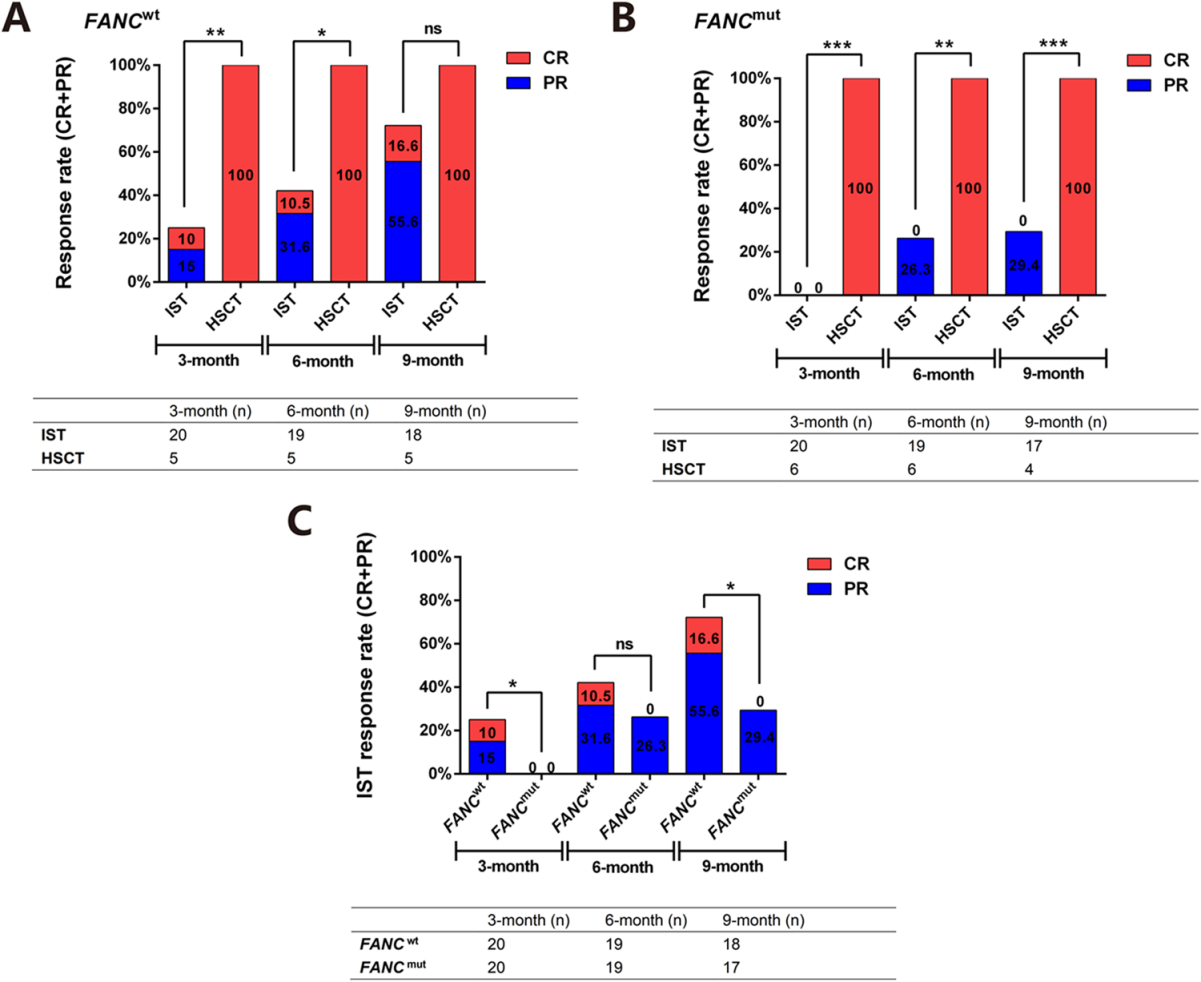
Comparison of IST vs. HSCT on response rate based on FANC status. All the patients were divided into two groups based on FANC mutation. The comparison of response rate (CR+PR) of patients (with or without FANC mutation, Figure 6A and 6B) to HSCT or IST, as well as IST response based on FANC mutation (Figure 6C) were compared based on 3, 6, 9 months observation. ns means no significant difference, *<0.05, **<0.01, ***<0.001. The number of patients included in each subgroup were shown

Based on *FANC* status, the response was further compared between different therapeutic methods, IST vs. HSCT. In the *FANC*^wt^ group, results showed that the efficiencies of IST were 25%, 42.1%, and 72.2% at the 3^rd^, 6^th^, and 9^th^ months, compared to all 100% in the HSCT group. And the difference was significant at the 3^rd^ and 6^th^ months (*P* = 0.005, *P* = 0.041), but not at the 9^th^ month (*P* = 0.280) (Fig. 6A). In the *FANC*^mut^ group, the results were 0%, 26.3%, and 29.4% in the IST group, compared to all 100% in the HSCT group at 3^rd^-, 6^th^-, and 9^th^-month evaluation, respectively. All had a significant difference (*P* < 0.001, *P* = 0.002, and *P* = 0.001, respectively) (Fig. 6B). When considering the effect of *FANC* status on IST or HSCT efficiency, we found that the *FANC* status did not influence the HSCT efficiency (all 100%), but in the IST, the 3^rd^-, 6^th^-, and 9^th^-month efficiencies were 25.0% vs. 0% (*P* =0 .017), 42.1% vs. 26.3% (*P* = 0.498), and 72.2% vs. 29.4% (*P* = 0.011), respectively, between *FANC*^wt^ and *FANC*^mut^ (Fig. 6C).

#### Comparison of different IST regimens on response rate based on FANC status

**Fig. 7:**
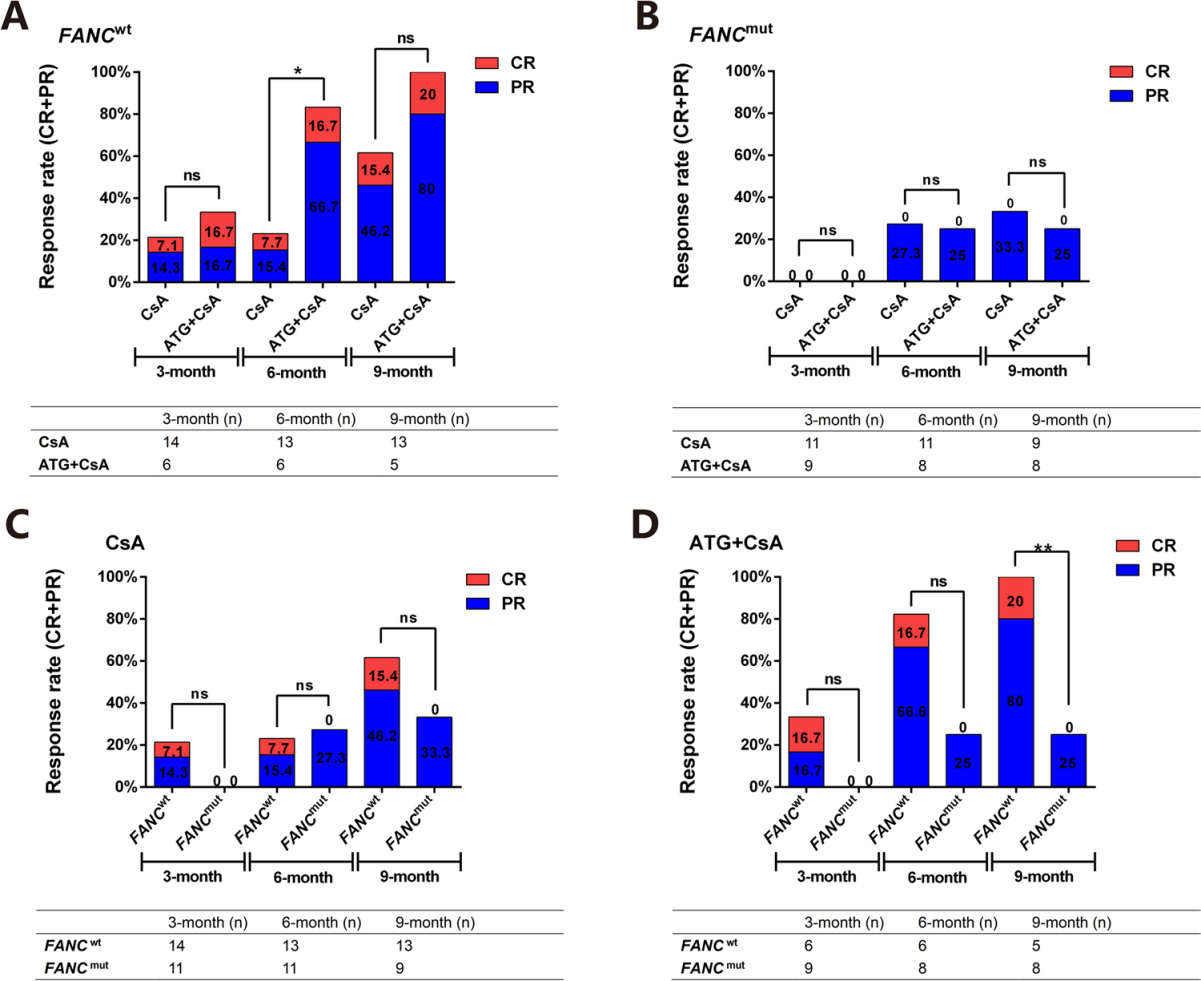
Comparison of different IST regimens on response rate based on FANC status. All the aplastic anemia patients received IST were divided into two groups based on FANC mutation. The comparison of response rate (CR+PR) of patients (with or without FANC mutation, Figure 7A and 7B) to CsA or ATG+CsA, as well as IST (CsA or ATG+CsA, Figure 7C and 7D) response based on FANC mutation were compared based on 3, 6, 9 months observation. ns means no significant difference, *<0.05. The number of patients included in each subgroup were shown

Twenty-four patients with *FANC*^wt^ status and 20 patients with *FANC*^mut^ status received IST, and the basic characteristics are summarized in Table 2. No significant differences were observed among age, gender, blood transfusion, and the severity of AA. The specific distribution according to the severity of AA is shown in Table 2. The WBC count, neutrophil count, HB, and absolute Ret count were much lower in the IST-*FANC*^mut^ group (*P* = 0.018, 0.018, 0.031, and 0.007, respectively). In the *FANC*^wt^ group, results showed that the efficiencies of CsA were 21.4%, 23.1%, and 61.6% at 3^rd^, 6^th^, and 9^th^ months, compared to 33.4%, 83.4%, and 100% in the ATG + CsA group. And the difference was only significant at the 6^th^-month evaluation (*P* = 0.041) (Fig. 7A). In the *FANC*^mut^ group, the results were 0%, 27.3%, and 33.3% in the CsA group, compared to 0%, 25.0%, and 25.0% in the ATG + CsA group at the 3^rd^-, 6^th^-, and 9^th^-month evaluation, respectively. And no statistical differences were observed (all *P* > 0.05) (Fig. 7B). When considering the effect of *FANC* status on CsA or ATG + CsA efficiency, we found that in the CsA single treatment group, the 3^rd^-, 6^th^-, and 9^th^-month efficiencies were 21.4% vs. 0% (*P* = 0.230), 23.1% vs. 27.3% (*P* = 1.0), and 61.6% vs. 33.3% (*P* =0.387), respectively; no statistical differences were observed between *FANC*^wt^ and *FANC*^mut^ (Fig. 7C). In the ATG + CsA group, the 3^rd^-, 6^th^-, and 9^th^-month efficiencies were 33.4% vs. 0% (*P* = 0.143), 83.3% vs. 25% (*P* = 0.103), and 100% vs. 25% (*P* = 0.003), respectively, and the difference was only significant at the 9^th^-month evaluation (Fig. 7D).

**Table 2:** Clinical features of AA patients with/without *FANC* mutation who received IST

	AA with FANC mutation (20, 45.45%)	AA without FANC mutation (24, 54.55%)	*X*^2^/*Z*	*P*
Age/median (range)	22 (9–33)	22 (14–40)	− 1.334	0.182
Depend on blood transfusion (number of cases (percentage))	18 (90%)	18 (45.45%)	0.796	0.372
Classification (disease severity)/(cases, %)	20	24	4.955	0.175
VSAA	3 (15%)	0		
SAA	4 (20%)	5 (20.83%)		
TD-NSAA	11 (55%)	13 (54.17%)		
NSAA	2 (10%)	6 (25%)		
CSA single treatment composition (cases, %)	11	18	1.173	0.556
SAA	1 (9.09%)	2 (11.11%)		
TD-NSAA	8 (72.73%)	10 (55.56%)		
NSAA	2 (18.18%)	6 (33.33%)		
ATG + CSA combined therapy composition (cases, %)	9	6	2.500	0.287
VSAA	3 (33.3%)	0		
SAA	3 (33.3%)	3 (50%)		
TD-NSAA	3 (33.3%)	3 (50%)		
WBC count (10^9^/L)/median (range)	2.05 (0.6–4.2)	2.7 (1.6–5.8)	− 2.360	**0.018**
Absolute neutrophil count (10^9^/L)/median (range)	0.65 (0.1–1.7)	1.15 (0.2–3.0)	− 2.375	**0.018**
Neutrophil < 0.5 10^9^/L (cases, %)	8 (40.00%)	5 (20.83%) 1.925	0.165	
HB (g/L)/median (range)	54.5 (28–102)	61.5 (43–124)	− 2.159	**0.031**
PLT count (10^9^/L)/median (range)	11.5 (1–36)	17 (4–68)	− 1.854	0.064
Absolute Ret count (10^9^/L, M)	2.465 (0.02–7.38)	3.445 (1.44–8.06)	− 2.711	**0.007**

Values in bold indicate *P*<0.05

*VSAA*: very severe aplastic anemia; *SAA*: severe aplastic anemia; *TD-NSAA*: transfusion-dependent none-severe aplastic anemia; *NSAA*: none-severe aplastic anemia; CsA:Cyclosporin A；*ATG*+*CsA*: antithymocyte globulin combined with Cyclosporin A；*WBC*: white blood cell, *RBC*: red blood cell; *HB*: hemoglobin; *PLT*: platelet

#### Comparison of immunological indexes based on FANC status

The WES analyses showed that besides the *FANC* mutation, the most coexisting mutations that may affect the immune system were *LIG4* (3/26), and the others include *TNFRS13B, AIRE, SMARCAL1, C8A, RFT1*, and *SERPING1* (1/26 in all). Considering the effect of immunological status on the treatment response in AA, we analyzed the lymphocytes and serum levels of immunoglobulins in peripheral blood before treatment. There were no statistical differences in IgA, IgG, IgM, Treg (CD4^+^CD25^+^CD127^-^), Th cells (CD3^+^CD4^+^), Ts cells (CD3^+^CD8^+^), and the ratio of CD4/CD8, and B cells (CD19^+^) among the *FANC* germline mutation subgroups (all *P* > 0.05) (Supplemental Table 3).

### Overall survival (OS) comparison based on therapy and FANC status

**Fig. 8:**
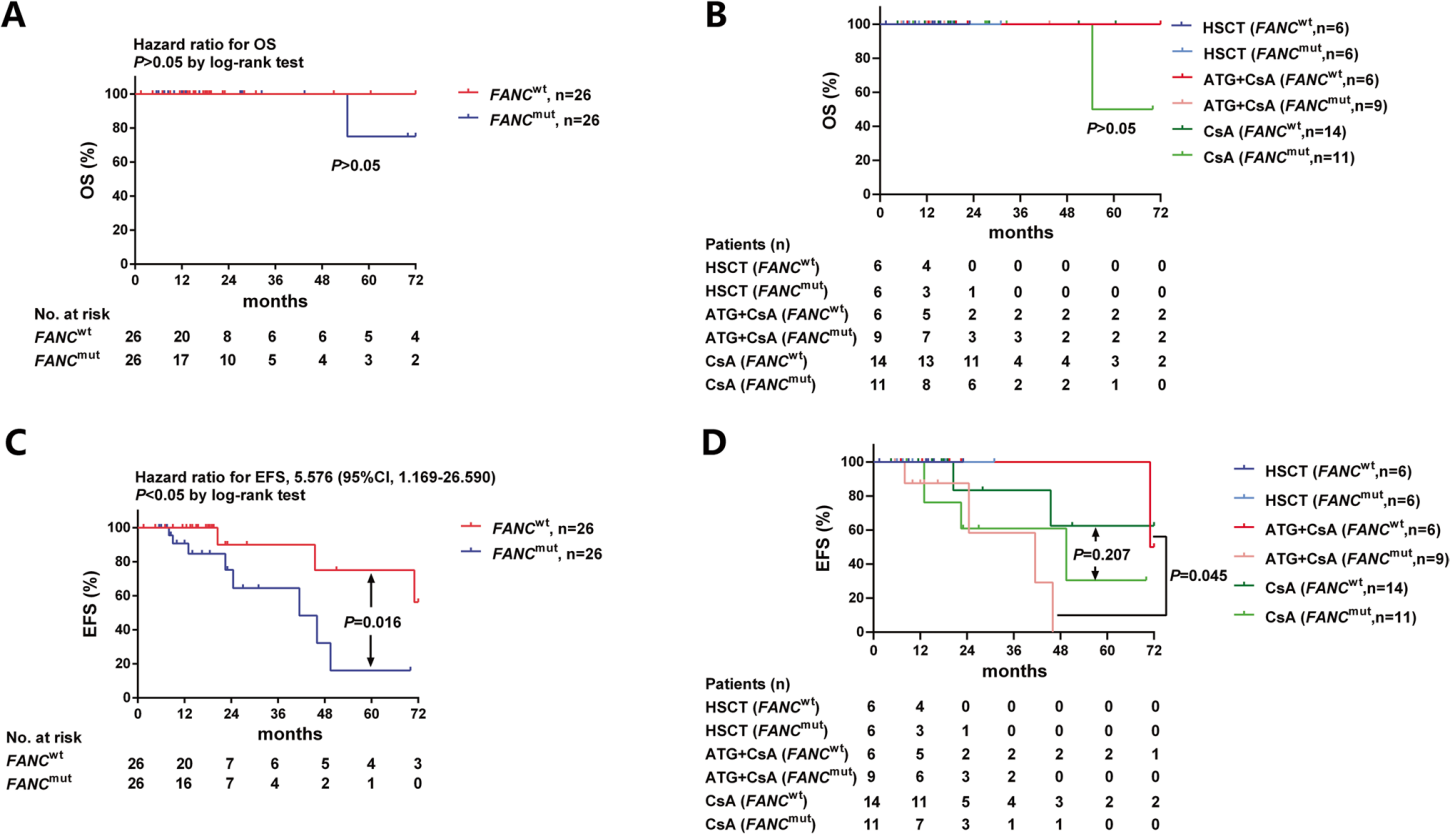
Overall survival (OS) and event free survival (EFS) comparison based on therapy and FANC status. The OS of entire population (Figure 8A) and different treatment regime (Figure 8B) based on FANC mutation were compared, and no statistic differences were observed. The EFS of entire population (Figure 8C) and different treatment regime (Figure 8D) based on FANC mutation were compared. The patients without FANC mutation (FANCwt) had a significantly better EFS than that with mutation (FANCmut) (P<0.05), and the ATG+CsA treatment group without FANC mutation showed a better EFS compared to that with FANC mutation (*P*<0.05)

We excluded 4 patients who were lost to follow-up and 5 patients who only received supportive treatment. Finally, 40 patients who received IST and 12 patients who received HSCT were eligible for OS analysis. There were respectively 20 and 6 patients who received IST or HSCT in the *FANC*^wt^ group and 20 and 6 patients who received IST or HSCT in the *FANC*^mut^ group. The median follow-up period was 18.25 (1.5–72) months. There was only one patient in the *FANC*^mut^ group whose condition transformed into acute myeloid leukemia (AML) 49 months after the failure of CsA single treatment and died due to severe infection. All others survived at the time of the last follow-up. The differences in overall survival in patients with or without *FANC* mutation were not present (*P* > 0.05) (Fig. 8A). And there were also no differences among different therapeutic methods (HSCT, ATG + CsA, or CsA, with or without *FANC* mutations) (Fig. 8B).

### Event-free survival (EFS) comparison based on therapy and FANC status

At a median follow-up period of 18.25 (1.5–72) months, 40 patients who received IST and 12 patients who received HSCT were eligible for the EFS analysis. In the *FANC*^wt^ group, two patients further received HSCT because of the failure from CsA single treatment (20.5 months and 45.5 months). Another one relapsed at 71 months after partial remission from ATG + CsA treatment and suffered from transfusion-dependent anemia and thrombocytopenia. For patients in the *FANC*^mut^ group, six patients switched treatments after preliminary treatment failure, including one that transformed into acute myeloid leukemia. All these results showed that the patients without *FANC* mutation had a better EFS (*P* = 0.016) (Fig. 8C), and the subgroup analysis showed that the differences were significant in patients received ATG + CsA treatment (*P* = 0.045) (Fig. 8D).

### EFS risk evaluation based on patients’ baseline characteristics

**Fig. 9:**
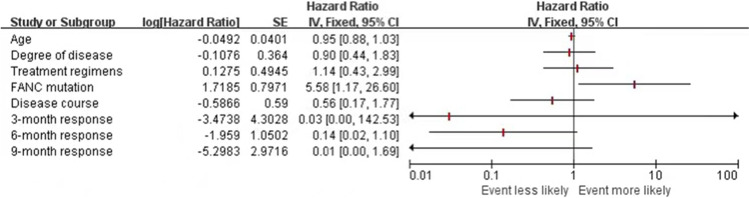
Event free survival (EFS) risk evaluation based on patients’ baseline characteristics. Age, degree of disease, treatment regimens, FANC mutation and 3, 6, 9-month response were entered into cox regression separately, results showed FANC mutation was the only predictor associated with poor EFS (*P*=0.031)

We used cox regression to identify the possible factors affecting the EFS. Firstly, all possible factors such as age, degree of disease, treatment regimens, *FANC* status, the interval time from diagnosis to treatment, and 3-, 6-, and 9-month response were included in cox regression separately, and then variables with a *P* value < 0.05 were added to the further cox regression (Table 3). The results showed that *FANC* mutation was the only predictor associated with poor EFS (odds ratio (OR) = 5.576, 95% confidence interval (CI) 1.169–26.590, *P* = 0.031) (Fig. 9).

**Table 3:** EFS risk evaluation

Factors	SE	95% CI	*P*
Age	0.040	0.952 (0.880–1.029)	0.214
Degree of disease	0.364	0.898 (0.440–1.834)	0.767
Treatment regimens	0.067	1.136 (0.431–2.997)	0.796
*FANC* mutation	0.797	5.576 (1.169–26.590)	**0.031**
Disease course*	0.590	0.555 (0.175–1.764)	*0.318*
3-month response	4.303	0.031 (0.000–142.544)	0.420
6-month response	1.052	0.141 (0.018–1.105)	0.062
9-month response	2.973	0.005 (0.000–1.692)	0.075

Values in bold indicate *P*<0.05

*The interval time from diagnosis to treatment

## Discussion

Acquired aplastic anemia has its unique clinical behavior, and recent advances have also shown more complicated phenotypes and imperfect genotype-phenotype correlations [26]. In China, quite a lot of patients with AA choose IST, because of age limitation, financial difficulties, unsuitable donors, and other reasons. In patients who are treated with IST, especially ATG + CsA, the response rate was not good as those reported in Western countries. This variation in response may be because of the unavailability of horse-ATG [27] and thus the species differences in ATG, which was not comprehensively elucidated. In addition, with the analysis of IST predictive response factors [4–6], there are still a considerable number of patients suffering from poor prognoses even if they have workable treatment.

With the widespread use of WES diagnostic techniques in the clinic, the number of patients benefiting from these tools has been rapidly increasing. The biallelic compromise of one of 22 *FANC* genes involving the FA/BRCA repair pathway caused Fanconi anemia (FA) with an incidence rate of 1 in 300,000 live births and a prevalence rate of 1–9 per million [28, 29], which was reported to be the most frequent inherited cause of bone marrow disorder (BMF) clinically characterized by congenital malformations [29, 30]. The *FANC* mutation carrier frequency varies in different regions. Generally, the mutation frequency of the *FANC* gene in a normal population is 1/189 and is increased to 1/64 in some populations with a high incidence of FA [31]. At present, there is no clinical report on acquired aplastic anemia complicated with *FANC* germline mutations and also no data report on the frequency of *FANC* mutations in Chinese. In this study, the WES analyses showed that there was an unexpectedly high rate of *FANC* germline mutations in the cohort (28/61, 45.9%), which was probably 96–160 times than the healthy population, denoting the significance of this exploration. Overall, genetic variants were found in most of the patients, with an average of 5 mutations per patient, but the proportion of *FANC* gene mutation was higher than that of other gene mutations in the whole-exome sequencing 11.73% (38/324) vs. 0.31–1.54% (1/324–5/324) (detailed gene mutation information is shown in supplemental Table 4).

Biallelic inactivation of the *FANC* gene results in the rare and autosomal recessive disease, Fanconi anemia, a disorder characterized by developmental anomalies, bone marrow failure, and susceptible to malignancies. In this study, all *FANC* mutated patients were confirmed negative for mitomycin-C-induced chromosome breakage tests and did not have any congenital malformations as well as family history. Besides, all of the FANC mutations identified in patients were heterozygous germline mutations, which may explain the lack of FA phenotype despite *FANC* mutations. Based on the baseline clinical analysis, we found that the patients with *FANC* mutation had a significantly lower level of ARC (*P* < 0.05), probably leading to more VSAA + SAA cases in the mutation group (50% vs. 33.3%, *P* = 0.205). More patients received IST than HSCT, and regarding the response rate of IST, CsA was comparable to ATG + CsA, but the response rate was much lower than HSCT (3-, 6-, and 9-month results). Further, subgroup analysis based on the *FANC* mutation showed that there was an earlier (3-month result) and a better (9-month result) IST response than that in the *FANC*^mut^ group. The patients with wide-type *FANC* had a much better response to ATG + CsA than CsA (but *P* > 0.05), while the *FANC* mutation eliminated the advantage of ATG + CsA (regardless of CsA or ATG + CsA, the response of < 40% at any time points of evaluation). The long-term outcome showed that OS was not influenced by therapeutic methods as well as *FANC* status; however, the EFS was significantly better in IST without *FANC* mutation than in cases with mutations. All the results indicated that *FANC* mutation is associated with the response and EFS of AA patients receiving ATG + CsA treatment, and HSCT treatment performed better in this population.

*FANC* mutation was involved in the regulation of hematopoietic function. The mechanism of bone marrow failure caused by *FANC* gene mutation remains to be elucidated. FA pathway coordinates DNA damage signaling and enzymatic DNA repair processes during DNA replication. Germline homozygous mutations in the FA genes led to deficiencies of FA DNA repair pathway, which was thought to be the main mechanism responsible for bone marrow failure in FA [32]: (a) HSC defect starts before birth, and mutant HSCs are unambiguously reduced during development resulting in stem cell apoptosis [32, 33]; (b) attrition of HSC pools caused by unresolved DNA damage-dependent cell cycle arrest and apoptosis [32]. Studies also found that non-canonical pathways mediated stem cell exhaustion. Many Fanconi anemia proteins [34] participated in control of signaling pathways that stress HSCs; they had additional functions in protecting cells from oxidative stress, inflammation, and endogenous aldehyde–induced death, as well as facilitating autophagy. When faced with inflammatory stress, *FANC* mutated HSCs will be hypersensitive, resulting in the overproduction of inflammatory cytokines [29]. Overexpressed TNF-α and IFN-γ inhibited erythropoiesis [35], increased TGF-β1 that arrested the pro-apoptotic state, increased regulatory T cells, reduced B/NK cells, and inhibited the growth and differentiation of early hematopoietic stem cells (HSCs) as well as IgG and IgM [36]. All these may be reasonable for poor IST efficacy in AA patients with FA mutation. However, these mechanisms were from the research on bone marrow failure caused by FA homozygous mutation. Recently, studies revealed a certain incidence of heterozygous *FANC* mutation in AA patients [37, 38], but the underlying mechanisms in bone marrow failure were not well explained. They speculated that the eventual onset of the disease originated from heterozygous *FANC* mutation was more complex and delayed and might relate to the accumulating chromosomal structural abnormalities, which implied the normal alleles are not entirely protective. In this study, we also explored the immunophenotype of peripheral blood lymphocytes and serum levels of immunoglobulins (Ig) that may be involved in the immune response, but there were no statistical differences. However, the patients with the *FANC* mutation had lower bone marrow CD34^+^ cells and more severe cases. We were not able to verify the mechanisms in this study, and more exploratory studies are needed to explain the contribution of heterozygous *FANC* mutation in the development of AA and its effect in lower IST response.

In conclusion, for patients younger than 40 years old without *FANC* germline mutation, ATG + CsA can exert a curative effect that was comparable to HSCT, but for cases with *FANC* mutation, HSCT can be a better choice. *FANC* germline mutation can negatively influence the treatment response with IST in Chinese aplastic anemia patients. The relatively small sample size is the limitation of our study; we will further initiate multi-center and larger sample size study.

## Supplementary information

ESM 1: Supplement Table 1. Clinical features of AA with germline FANC mutation (DOC 67 kb)

ESM 2: Supplement Table 2. Distribution of therapeutic methods of AA patients with/without FANC mutation (DOC 30 kb)

ESM 3: Supplement Table3. Comparison of immunological status between AA patients with/without FANC mutation who received IST (DOC 34 kb)

ESM 4: Supplemental Table 4. Detailed Gene Mutation distributions of all the AA patients (DOC 60 kb)

## Data Availability

The data used and/or analyzed during the current study are available from the corresponding author upon a reasonable request.
