# Supplementary material for: Whole-exome sequencing identifies FANC heterozygous germline mutation as an adverse factor for immunosuppressive therapy in Chinese aplastic anemia patients aged 40 or younger: a single-center retrospective study
Source: Ann Hematol. 2023 Jan 9;102(3):503–17. doi: 10.1007/s00277-023-05086-9 (PMC9977704; doi:10.1007/s00277-023-05086-9)
Supplement: Supplementary file 1 — Supplement Table 1. Clinical features of AA with germline FANC mutation (DOC 67 kb) [file 277_2023_5086_MOESM1_ESM.doc]

**Supplement Table 1. Clinical features of AA with germline FANC mutation**

| Name ID# | Age with hematology abnormality/Gender | Age diagnosed with AA | Transfusion dependent (Y/N) | Genotyping  Specimens (BM/PB; OM/Nail) | FANC Germline Mutation type/ *Clinical interpretation* | Chromosomal location | Family germline gene verification | Treatment | Curative effect | Prognosis |
| --- | --- | --- | --- | --- | --- | --- | --- | --- | --- | --- |
| P01 | 15/M | 15 | Y | BM; OM | BRCA2,exon11,c.G2968T,p.E990X/  *likely benign*; FANCA,exon26,c.C2500G,p.L834V/  *likely benign*;  FANCA,exon23,c.C2140T,p.R714W/  *uncertain significance*;  PALB2,exon9,c.G2968T,p.E990X/  *pathogenic* | chr13:32914640  chr16:89836249  chr16:89838097  chr16:23634318 | Untested | Haplo-HSCT | CR | alive |
| P02 | 16/M | 16 | Y | BM; OM | FANCC,exon8,c.C763A,p.L255M/  *uncertain significance*;  FANCA,exon23,c.G2080A,p.D694N/  *uncertain significance* | chr9:97897708  chr16:89838157 | Untested | Supportive care | NR | alive |
| P03 | 27/M | 27 | N | BM; OM | FANCA,exon26,c.G2428T,p.A810S/  *likely benign*; | chr16:89836321 | Untested | CsA | PR | alive |
| P04 | 30/M | 30 | Y | BM; OM | FANCA,exon22,c.2003G>T,p.S668I/  *likely benign*;  ERCC4, exon9,c.1900A>G,p.I634V/  *uncertain significance* | chr16:89839690  chr16:14031711 | Untested | ATG | PR | alive |
| P05 | 7/F | 12 | Y | BM; OM | FANCA,exon3,c.209A>G,p.K70R/  *uncertain significance* | chr16:89881002 | Father with FANCA,exon3 | Haplo-HSCT | CR | alive |
| P06 | 8/M | 28 | Y | BM; Nail | FANCB,exon3,c.440T>C,p.V147A/  *uncertain significance* | chrX:14883193 | Untested | CsA | NR  at the 3rd month | alive |
| P07 | 21/F | 29 | Y | BM; OM | FANCD2,exon41,c.C3973A,p.L1325M/  *uncertain significance*;  FANCD2,exon25,c.A2309G,p.K770R/  *uncertain significance* | chr3:10136893  chr3:10107587 | Father with FANCD2,exon41; Mother with FANCD2,exon25 | Haplo-HSCT | CR | alive |
| P08 | 23/M | 23 | Y | BM; OM | FANCD2,exon29,c.2723C>T,p.T908I/  *likely benign*; | chr3:10116221 | Untested | CsA | PR | alive |
| P09 | 20/F | 20 | Y | BM; OM | FANCE,exon2,c.C598T,p.R200C/  *uncertain significance* | chr6:35423873 | Father with FANCE | Haplo-HSCT | CR | alive |
| P10 | 17/M | 18 | Y | BM; OM | FANCE,exon2,c.C316T,p.R106W/  *uncertain significance* | chr6:35423591 | Untested | Haplo-HSCT after CsA failure | CR | alive |
| P11 | 20/M | 20 | Y | PB; Nail | FANCF,exon1,c.G182T,p.R61L/  *uncertain significance* | chr11:22647175 | Untested | CsA | NR at the 5^th^ month,less than 9 months | alive |
| P12 | 18/M | 18 | Y | BM; OM | FANCG,exon1,c.A55G,p.K19E/  *uncertain significance* | chr9:35079467 | Untested | ATG after CsA failure | NR | alive |
| P13 | 3/F | 10 | Y | BM; OM | FANCL,exon7,c.G490A,p.D164N/  *uncertain significance;*  SLX4,exon15, c.G5239A , p.V1747M/  *uncertain significance* | chr2:58198644;  chr16:3582608 | Father negative | Haplo-HSCT after ATG failure | CR | alive |
| P14 | 26/F | 28 | Y | BM; OM | FANCM,exon14,c.G3469A,p.E1157K/  *uncertain significance* | chr14:45176223 | Untested | CsA | NR | alive |
| P15 | 3/F | 11 | Y | BM; OM | PALB2,exon13,c.T3379C,p.C1127R/  *uncertain significance*;  PALB2,exon4,c.A925G,p.I309V/  *likely benign* | chr16:23614962  chr16:23646942 | Untested | Supportive care | NR | alive |
| P16 | 11/M | 11 | Y | BM; Nail | SLX4,exon12,c.A2381T,p.D794V/  *uncertain significance* | chr16:3641258 | Mother with SLX4; Father negative | CsA | PR | alive |
| P17 | 15/M | 26 | Y | BM; OM | SLX4,exon14,c.C4765T,p.R1589C/  *uncertain significance* | chr16:3633486 | Father with SLX4 | Haplo-HSCT after 2rd ATG failure | CR | alive |
| P18 | 15/F | 25 | Y | BM; OM | FANCE,exon5,c.T971C,p.M324T/  *uncertain significance*;  BRCA2,exon10,c.T1773G,p.I591M/  *uncertain significance*;  RPL3L,exon2,c.C165G,p.H55Q/  *uncertain significance* | chr6:35426075  chr13:32907388  chr16:2003988 | Untested | Haplo-HSCT after ATG failure | CR | alive |
| P19 | 6/F | 9 | N | BM; OM | FANCL,exon5,c.C335T,p.S112L/  *uncertain significance* | chr2:58221981 | Untested | CsA | PR | alive |
| P20 | 11/M | 16 | Y | BM; OM | BRCA2,exon11,c.C2260A,p.Q754K/  *likely benign* | chr13:32910752 | Untested | CsA | NR | Progress into leukemia at 49 months /died |
| P21 | 38/M | 38 | Y | BM; OM | FANCI,exon21,c.A2011G,p.I671V/  *likely benign*;  FANCI,exon24,c.A2604C,p.E868D/  *likely benign* | chr15:89835937  chr15:89838293 | Untested | Haplo-HSCT | CR | alive |
| P22 | 22/M | 33 | Y | BM; OM | FANCA,exon30,c.A2944G,p.T982A/  *likely benign* | chr16: 89825022 | Untested | Haplo-HSCT after ATG failure | CR | alive |
| P23 | 7/F | 9 | Y | BM; OM | PALB2,exon5, c.2474G>C,p.R825T/  *uncertain significance*  ERCC4,exon8,c.1787C>A,p.A596E/  *uncertain significance* | chr16:23641001  chr16:14029576 | Untested | Haplo-HSCT | CR | alive |
| P24 | 14/F | 14 | Y | BM; Nail | BRIP1,exon19,c.2830C>G,p.Q944E/  *likely benign* | chr17:59763272 | Mother with BRIP1; Father negative | ATG | NR | alive |
| P25 | 20/F | 21 | Y | BM; OM | SLX4,exon2,c.437C>T,p.S146F/  *uncertain significance* | chr16:3658529 | Untested | ATG | NR at the 9^th^ month | alive |
| P26 | 22/F | 22 | Y | BM; OM | UBE2T, exon2, c.G71A,p.C24Y/  *uncertain significance* | chr1:202335684 | Untested | Haplo-HSCT | CR | alive |
| P27 | 25/F | 19 | Y | BM; OM | BRCA2,exon11,c.5200G>A,p.E1734K/  *uncertain significance*  SLX4,exon12,c.2854_2855delinsAT,p.A952M/  *uncertain significance* | chr13: 32913692  chr16: 3640784 | Untested | CsA | NR | alive |
| P28 | 25/M | 25 | Y | BM; OM | FANCD2,exon43,c.4234_4239del ,p.E1411_S1412del/  *uncertain significance* | chr3: 10140452 | Untested | ATG | NR at the 3th month,less than 6 months | alive |

Note: all the FANC germline mutations were confirmed heterozygous. CSA: cyclosporine; ATG: antithymocyte globulin; HSCT: hematopoietic stem cell transplantion; OM: oral mucosa
