# Supplementary material for: Whole-exome sequencing identifies FANC heterozygous germline mutation as an adverse factor for immunosuppressive therapy in Chinese aplastic anemia patients aged 40 or younger: a single-center retrospective study
Source: Ann Hematol. 2023 Jan 9;102(3):503–17. doi: 10.1007/s00277-023-05086-9 (PMC9977704; doi:10.1007/s00277-023-05086-9)
Supplement: Supplementary file 2 — Supplement Table 2. Distribution of therapeutic methods of AA patients with/without FANC mutation (DOC 30 kb) [file 277_2023_5086_MOESM2_ESM.doc]

**Supplement Table 2. Distribution of therapeutic methods of AA patients with/without FANC mutation**

**Note:** CsA: cyclosporine; ATG+CsA: antithymocyte globulin with cyclosporine; HSCT: hematopoietic stem cell transplantion.

|  | AA with FANC mutation  (28,45.90%) | AA without FANC mutation  (33,54.10%) | X^2^ | *P* |
| --- | --- | --- | --- | --- |
| Therapy  (Number of cases (percentage)) |  |  | 2.094 | 0.553 |
| CsA | 11(39.29%) | 18(54.55%) |  |  |
| ATG+CsA | 9(32.14%) | 6(18.18%) |  |  |
| HSCT | 6(21.43%) | 6(18.18%) |  |  |
| Supportive care | 2(7.14%) | 3(9.09%) |  |  |
