# Supplementary material for: Whole-exome sequencing identifies FANC heterozygous germline mutation as an adverse factor for immunosuppressive therapy in Chinese aplastic anemia patients aged 40 or younger: a single-center retrospective study
Source: Ann Hematol. 2023 Jan 9;102(3):503–17. doi: 10.1007/s00277-023-05086-9 (PMC9977704; doi:10.1007/s00277-023-05086-9)
Supplement: Supplementary file 3 — Supplement Table3. Comparison of immunological status between AA patients with/without FANC mutation who received IST (DOC 34 kb) [file 277_2023_5086_MOESM3_ESM.doc]

**Supplement Table3. Comparison of immunological status between AA patients with/without FANC mutation who received IST**

|  | AA with FANC mutation  (20,45.45%) | AA without FANC mutation  (24,54.55%) | Z | *P* |
| --- | --- | --- | --- | --- |
| Total serum immune globulin (g/L)/ Median (range) | 19.9(13.8-32.2) | 23.2(15.4-34.5) | -1.681 | 0.093 |
| IgA | 1.6(0.72-2.64) | 1.78(0.51-3.81) | -0.048 | 0.962 |
| IgG | 7.92(6.4-14.45) | 10.5(5.67-15.1) | -1.717 | 0.086 |
| IgM | 1.42(0.48-2.49) | 1.07(0.62-3.9) | -0.690 | 0.490 |
| CD3^+^CD45^+^ (%)/ Median (range) | 78.59(58.7-88.85) | 78.75(62.22-91.26) | -0.143 | 0.886 |
| CD3^+^CD4^+^ (% of CD3^+^CD45^+^) / Median (range) | 38.295(9.93-56.3) | 39.00(24.3-60.52) | -0.542 | 0.587 |
| CD3^+^CD8^+^ (% of CD3^+^CD45^+^) / Median (range) | 35.63(17.41-63.15) | 33.04(19.96-49.89) | -0.971 | 0.332 |
| CD4^+^CD25^+^CD127^-^ (% of CD4^+^CD25^+^)/ Median (range) | 5.35(2.5-9.7) | 5.36(2.8-8.2) | -0.271 | 0.786 |
| CD4^+^/CD8^+^ (ratio)/ Median (range) | 1.071(0.157-3.234) | 1.163(0.533-3.032) | -0.999 | 0.318 |
| CD19^+^ (% of all lymphocytes,Median ) | 11.470(1.63-24.21) | 11.445(4.19-24.59) | -0.340 | 0.734 |
