# Supplementary material for: Whole-exome sequencing identifies FANC heterozygous germline mutation as an adverse factor for immunosuppressive therapy in Chinese aplastic anemia patients aged 40 or younger: a single-center retrospective study
Source: Ann Hematol. 2023 Jan 9;102(3):503–17. doi: 10.1007/s00277-023-05086-9 (PMC9977704; doi:10.1007/s00277-023-05086-9)
Supplement: Supplementary file 4 — Supplemental Table 4. Detailed Gene Mutation distributions of all the AA patients (DOC 60 kb) [file 277_2023_5086_MOESM4_ESM.doc]

**Supplemental Table 4. Detailed Gene Mutation distributions of all the AA patients**

|  | **Gene mutation distributions** |
| --- | --- |
| **AA patients with *FANC* mutation** | |
| P01 | *BRCA2, FANCA, PALB2* |
| P02 | *FANCC, FANCA, PROC* |
| P03 | *FANCA, ATRX, CD36* |
| P04 | *FANCA, FAT1, KDM4C, KMT2C, TET2, MN1, NCOR2, PTPRD, RPS24, UNC13D* |
| P05 | *FANCA, ATXN7L1, CDK12, CEBPA, ERCC4, FAT1, KDM4C, KMT2C, MN1, NCOR2, PTPRD, RPS24, UNC13D* |
| P06 | *FANCB, KMT2C, ABCB1, ATM, FBXO31, GABRA6, UGT1A6,WDR90* |
| P07 | *FANCD2* |
| P08 | *FANCD2, ARID1B, BARD1, BCL11B, CIC, DUSP2, LYST, PRKDC, STAT6, UNC13D* |
| P09 | *FANCE, EPPK1, ETV6, LIG4* |
| P10 | *FANCE, RPL29, LRBA, NFKB2, ATM, MSH6, HPS5, RFT1, LDHA, PCCA, HK3, EPG5* |
| P11 | *FANCF* |
| P12 | *FANCG, PS10, RPS6KB2* |
| P13 | *FANCL, SLX4, AIRE* |
| P14 | *FANCM, BCOR, PANK2, SMARCAL1* |
| P15 | *PALB2, BCOR, BTK, RPL3L* |
| P16 | *SLX4, ACTN1, PFKFB1, PLCG2, SLC22A17,DOCK8, LPL* |
| P17 | *SLX4, ACVRL1, AK2, CTC1, CUBN, ABCB10, C8A, RFT1, F2, SERPING1, ZFPM1, APC, ARID1A* |
| P18 | *FANCE, BRCA2, RPL3L* |
| P19 | *FANCL, RPS6KA2,GAA* |
| P20 | *BRCA2* |
| P21 | *FANCI, GJB2* |
| P22 | *FANCA, RPS3, ADAMTS13, LBR* |
| P23 | *PALB2, ERCC4, ASXL2, BARD1, CCT6B, DAXX, FAT1, FGFR4, KMT2C, LYST, NUP214, STAT5A, WDR90* |
| P24 | *BRIP1, ACTR5, ANKRD26, ASXL1, CD101, FGF3, KMT2D, SGK1, TNFAIP3* |
| P25 | *SLX4, DNMT3B, KAT6A, NCOR2, P2RY8, RECQL4, SLX4, UNC13D* |
| P26 | *UBE2T, HEPH, TET2* |
| P27 | *BRCA2, SLX4, FLT1, JAK1, KDM2B, MSH3, MTRR, PRF1, PRKDC, SDHB, SLC28A3* |
| P28 | *FANCD2, AKT1, KMT2D, NPC1, PLCG1, TNFRSF14* |
| **AA patients without *FANC* mutation** | |
| P01 | *CBLB, CUX1, EPHA3, JAK2, KAT6A, PIGA, SDHA* |
| P02 | *ANK1* |
| P03 | *EGFR, GSK3B, KMT2C, NOTCH1, ROBO2* |
| P04 | *SLCO1B1* |
| P05 | *PIGT, CHD7, PPOX, STEAP3, ARNT, NLRP12, SP110 , PYGL, GPRC5A, CD36, NR3C2 , ESCO2, KIT* |
| P06 | *NBN, LIG4, TCF3* |
| P07 | *MAGT1, RTEL1, PHKA1, TMPRSS6, CUBN, ADAMTS13, HPS4, LAMB4, PFKP, CYP2C9* |
| P08 | *IL6, LBR, TFRC* |
| P09 | *BLM, CCT6B, CD36 , DDX18, GRM3, TCF3, ZNF703* |
| P10 | *HIST1H1E, ROS1, CSMD1, PDCD11* |
| P11 | *SPTB, PIEZO1* |
| P12 | *ETS2* |
| P13 | *CTPS1, PDCD11, SETBP1, SMPD1, SPEN, TCF4, TET3* |
| P14 | *SAMD9* |
| P15 | *ATR, GATA2, PIK3CG, TBX3, TET3* |
| P16 | *CIC, CSMD1, CSMD1, FGF6, PTPN1* |
| P17 | *RPL3, WIPF1, VWF* |
| P18 | *BCR, HNF1A, KDM5C, RET, ROS1, TLL2* |
| P19 | *EPB42, ASXL1, ATM, SH2B3* |
| P20 | *RPL3L* |
| P21 | *APC, BTLA, CBL, CEBPA, DAXX, FAT1, FOXP1, MKI67, NCOR1, NOTCH1, PCLO, PRKDC* |
| P22 | *LIG4* |
| P23 | *PRKDC* |
| P24 | *AMER1, CYP1A1, FAT1, HIPK2, NUP214, SPTA1* |
| P25 | *RTEL1, BCOR, EIF2B3, ANK1, JAK2, C8B, PIEZO1, LARS* |
| P26 | *LYST, THBD, NLRP3* |
| P27 | *CIC, CTC1, ELF1, GRM3, HIST1H1C, KDM5A, LAMP1, LRP1B, MN1, MSH6, MYO5A, MYOM2, NOTCH1, PDCD11, SMARCA1* |
| P28 | *KLF1, DNAJC21, ASXL1, MPL* |
| P29 | *PIEZO1, RBBP8* |
| P30 | *ASMTL , CHD2, CHD8, MUTYH, NCOR2, PIK3C2B, PTPRD, PTPRO, TET3, TSC1, TSC2, TSHR* |
| P31 | *EPHB1, ERBB2* |
| P32 | *RPS24* |
| P33 | *CUBN, UNC13D, SLC4A1* |
